# Discriminative Measurement of Absorbed Dose Rates in Air from Natural and Artificial Radionuclides in Namie Town, Fukushima Prefecture

**DOI:** 10.3390/ijerph18030978

**Published:** 2021-01-22

**Authors:** Koya Ogura, Masahiro Hosoda, Yuki Tamakuma, Takahito Suzuki, Ryohei Yamada, Ryoju Negami, Takakiyo Tsujiguchi, Masaru Yamaguchi, Yoshitaka Shiroma, Kazuki Iwaoka, Naofumi Akata, Mayumi Shimizu, Ikuo Kashiwakura, Shinji Tokonami

**Affiliations:** 1 Graduate School of Health Sciences, Hirosaki University, 66-1 Hon-cho, Hirosaki, Aomori 036-8564, Japan; kogura@hirosaki-u.ac.jp (K.O.); m_hosoda@hirosaki-u.ac.jp (M.H.); tamakuma@hirosaki-u.ac.jp (Y.T.); suzuki-takahito@fujielectric.com (T.S.); yamada.ryohei@jaea.go.jp (R.Y.); h20gg204@hirosaki-u.ac.jp (R.N.); r.tsuji@hirosaki-u.ac.jp (T.T.); masarun@hirosaki-u.ac.jp (M.Y.); ikashi@hirosaki-u.ac.jp (I.K.); 2 Institute of Radiation Emergency Medicine, Hirosaki University, 66-1 Hon-cho, Hirosaki, Aomori 036-8564, Japan; akata@hirosaki-u.ac.jp (N.A.); mshimizu@hirosaki-u.ac.jp (M.S.); 3 Faculty of Education, University of the Ryukyus, 1 Senbaru, Nishihara-cho, Okinawa 903-0213, Japan; y_shiro@cs.u-ryukyu.ac.jp; 4 National Institutes for Quantum and Radiological Science and Technology, 4-9-1 Anagawa, Inage, Chiba 263-0024, Japan; iwaoka.kazuki@qst.go.jp

**Keywords:** Fukushima Daiichi Nuclear Power Plant, Namie Town, natural radionuclides, artificial radionuclides, cesium-134, cesium-137, external exposure dose evaluation

## Abstract

Ten years have elapsed since the accident at the Fukushima Daiichi Nuclear Power Plant in 2011, and the relative contribution of natural radiation is increasing in Fukushima Prefecture due to the reduced dose of artificial radiation. In order to accurately determine the effective dose of exposure to artificial radiation, it is necessary to evaluate the effective dose of natural as well as artificial components. In this study, we measured the gamma-ray pulse-height distribution over the accessible area of Namie Town, Fukushima Prefecture, and evaluated the annual effective dose of external exposure by distinguishing between natural and artificial radionuclides. The estimated median (range) of absorbed dose rates in air from artificial radionuclides as of 1 April 2020, is 133 (67–511) nGy h^−1^ in the evacuation order cancellation zone, and 1306 (892–2081) nGy h^−1^ in the difficult-to-return zone. The median annual effective doses of external exposures from natural and artificial radionuclides were found to be 0.19 and 0.40 mSv in the evacuation order cancellation zone, and 0.25 and 3.9 mSv in the difficult-to-return zone. The latest annual effective dose of external exposure discriminated into natural and artificial radionuclides is expected to be utilized for radiation risk communication.

## 1. Introduction

**Figure 1:**
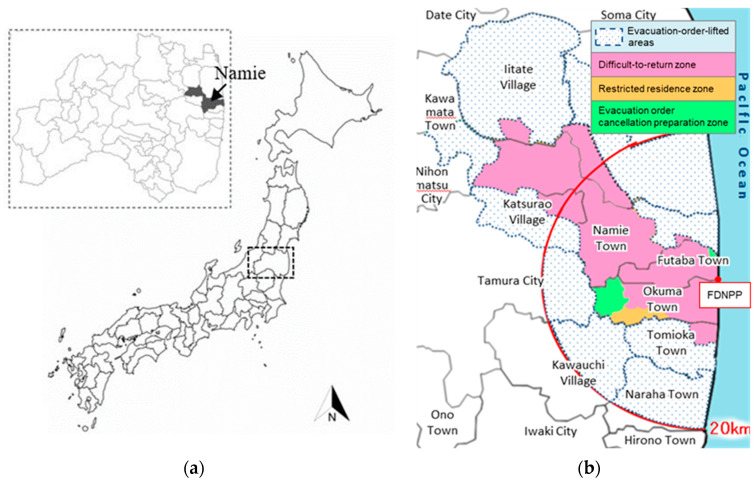
(**a**) Location of Namie Town, Fukushima Prefecture, Japan, and (**b**) officially designed evacuation zones as of 1 April 2017. (**a**) is created by d-maps.com (https://d-maps.com/carte.php?num_car=29487, https://d-maps.com/carte.php?num_car=11273). (**b**) is taken from the official website with permission from the administrative officer in Fukushima Prefecture [5].

On 11 March 2011, a magnitude 9.0 earthquake struck the Tohoku region along the eastern coast of Japan. The earthquake caused a tsunami with a height of more than 15 m, and affected the Fukushima Daiichi Nuclear Power Plant (FDNPP). The FDNPP lost power and the cores of Units 1 to 3 became heated and melted. This caused a hydrogen gas explosion [1]. As a result of the FDNPP accident, ^132^Te, ^131^I, ^134^Cs, ^137^Cs, and rare gases such as ^133^Xe, etc., were released into Fukushima Prefecture and other eastern regions of Japan [2]. The radioactivity of radionuclides released into the atmosphere is shown in the UNSCEAR 2013 report (Table 1) [3]. On the day of the accident, the Japanese government issued an indoor evacuation order to residents within 10 km of the FDNPP, and issued an evacuation order to residents within 20 km the next day [4]. Thereafter, the area where the annual cumulative dose may have exceeded 20 mSv, outside the 20 km area from the FDNPP was designated as a “planned evacuation zone”. In addition, regardless of the annual cumulative dose, the area within 20 to 30 km of the FDNPP was designated as an “emergency evacuation preparation zone” and the area within 20 km was designated as a “warning zone” [5]. Namie Town, Fukushima Prefecture (The location map that is shown in Figure 1a was made by original maps from d-maps.com), is also one of the areas significantly contaminated by radionuclides due to the FDNPP accident, and because it was a planned evacuation zone, the townspeople living there were forced to evacuate. In 2012, the area where the annual cumulative dose was confirmed to be 20 mSv or less was designated as an “evacuation order cancellation preparation zone”. This is the area where temporary return homes, restricted businesses such as shops, hospitals, and farming are permitted. Areas where the annual cumulative dose may exceed 20 mSv but are confirmed to be 50 mSv or less have been designated as a “restricted residence zone” and it has become possible to temporarily return home or enter for road restoration. Areas where the annual cumulative dose exceeds 50 mSv and the annual cumulative dose may not fall below 20 mSv, five years from 2012, has been designated as a “difficult-to-return zone”. Figure 1b indicates each area division, and taken from the official website of Fukushima Prefecture [5]. Subsequently, the artificial decontamination of radionuclides was actively promoted, and in 2017, six years after the earthquake, evacuation orders were lifted in some areas of Namie Town [6]. Currently, the return of evacuees is progressing, and by the end of November 2020, more than 1500 people were living in Namie Town [7]. Before the Great East Japan Earthquake, the registered population of Namie Town was 21,434 [8]. Years after the FDNPP accident, the returning residents continue to have a significant amount of radiation anxiety [9]. Experts in radiation science and psychology at each Japanese support organization, including the university of the current authors, have communicated radiation risk, and interacted with residents to reduce anxiety about radiation. In consideration of this, Kudo et al. conducted a questionnaire survey on the basic knowledge of radiation among those who returned to Namie Town. It was found that many Namie townspeople recognize that natural and artificial radiation have different effects on the human body, even if the effective dose is the same [10].

Since the FDNPP accident, national staff and researchers at universities and research institutions have been evaluating artificial radioactive contamination and investigating the distribution of ambient dose equivalent rates [11,12,13]. In addition, internal and external exposures from artificial radionuclides are being evaluated [14,15,16,17,18,19], and monitoring posts are installed in various locations to continuously measure the ambient dose equivalent rate [20]. In 2017, Shiroma et al. conducted a car-borne survey in Namie Town, Fukushima Prefecture, and reported that the absorbed dose rate in air was 0.041–11 µGy h^−1^ [21]. More than nine years have passed since the FDNPP accident, and the relative contribution of natural radiation to ambient dose equivalent rates is increasing because the dose of artificial radiation is decreasing. This means that it is not possible to estimate the effects on the human body due to artificial radionuclides, without correctly evaluating the dose from natural radionuclides. People with a high risk of internal exposure, such as agricultural workers, need information on internal exposure due to inhalation of dust. However, clarifying the actual conditions of external exposure from natural and artificial radionuclides is useful for radiation risk communication for general population, which has a low risk of internal exposure. In this study, the gamma-ray pulse-height distribution was measured and analyzed in Namie Town, which was divided into 1 km × 1 km meshes. An absorbed dose rate map that discriminated between natural and artificial radionuclides was created from the absorbed dose rate in the air, and the annual effective dose to external exposure was calculated.

## 2. Materials and Methods

### 2.1. Measurement Location and Method of γ-Ray Pulse-Height Distribution

From 15 September 2016 to 13 December 2019, gamma-ray pulse-height distributions were obtained at the 130 accessible points that divided the entire area of Namie Town into a mesh of 1 km × 1 km. A 3 × 3-inch NaI(Tl) scintillation spectrometer (EMF-211, EMF Japan Co., Himeji, Japan [22]) was used to obtain the measurements. The detector was installed 1 m above the ground and connected to a control laptop PC. The measurement time was 900 s. Latitude and longitude coordinate data were obtained using a Global Positioning System to create an absorbed dose rate map. Gamma-ray pulse-height distributions at 2–5 points were additionally acquired in six of the 130 meshes, and the fluctuation of the absorbed dose rate in air in the mesh was evaluated.

### 2.2. Analysis of Gamma-Ray Pulse-Height Distribution and Correction of Absorbed Dose Rate in Air

The gamma-ray pulse-height distributions obtained by the NaI(Tl) scintillation spectrometer is different from the distributions of the gamma-ray energy spectrum. The pulse-height distributions of gamma-ray are unfolded into the energy spectrum by a response matrix of 49 rows × 49 columns, and then the dose contributions for each radionuclide are calculated according to the previous reports to discriminate between natural and artificial radionuclides [23,24,25]. The absorbed dose rate in air obtained by the analysis needs to be corrected to consider the number of days elapsed from the measured date. Factors that reduce radioactivity in the environment include the physical half-life of radionuclides, diffusion by wind, rain, and infiltration into soil, and the implementation of artificial decontamination of radioactive substances. In order to comprehensively evaluate the factors that affect the attenuation of radioactivity, the apparent half-life was calculated using the data of the air dose rate that is regularly observed at the monitoring posts widely installed in Namie Town. There are 103 monitoring posts in Namie Town, and the measurement data are published on the website [20]. Some of these datasets have long-term data loss within the period in which we measured the gamma-ray pulse-height distribution, and significant dose increases and decreases in a short period of time that are not due to artificial decontamination. It is probable that the data loss could not be measured due to maintenance of the monitoring posts. The short-term significant fluctuation of the ambient dose equivalent rate may be due to a device malfunction, but the specific cause is unknown. These data may affect the appropriate time decay correction of absorbed dose rates in air. Therefore, the apparent half-life was calculated using the data of 55 monitoring posts, and excluding the lossy dataset and coefficient of determination R^2^ of less than 0.7 (not due to artificial decontamination) in the exponential approximation of the ambient dose equivalent rate. Equation (1) was used to calculate the apparent half-life (*T_a_*).

(1) $$T_{a}=t\;\times \;\frac{0.693}{ln\left(\frac{D_{1}}{D_{0}}\right)}$$

where *D*_0_ and *D*_1_ are the ambient dose equivalent rates (µSv h^−1^) as of 1 April 2016, and 1 April 2020, respectively, and t is the elapsed time, which was taken as used four years. The FDNPP accident released short half-life radionuclides such as ^131^I and ^133^Xe and long half-life radionuclides such as ^134^Cs and ^137^Cs. Originally, it was necessary to calculate the apparent half-life for each of the short-half-life and long-half-life radionuclides, but now that nine years have elapsed since the accident, the contribution from the short-half-life radionuclides can be ignored [26,27]. The apparent half-life was calculated using the simple formula in Equation (1), considering only the contribution from radionuclides with a long half-life. The calculated apparent half-life was divided into an evacuation order cancellation zone and a difficult-to-return zone, and the fluctuation was evaluated to examine the application to the correction of the absorbed dose rate in air.

### 2.3. Estimating the Effective Dose of External Exposure

The annual effective dose of external exposure in Namie Town was estimated using Equation (2), and the time-corrected absorbed dose rate in air.

*E* = *D* × *DCF* × *T* × (*Q*_*in*_ × *R* + *Q*_*out*_) (2)

where *D* is the time-corrected absorbed dose rate in air (nGy h^−1^) and *DCF* is a dose conversion factor (Sv Gy^−1^) from the absorbed dose rate in air to the effective dose to external exposure. The natural radionuclide component *DCF* uses 0.748, as reported by Moriuchi et al., and the artificial radionuclide uses 0.73, as reported by Omori et al. [28,29]. *T* is the number of hours per year, which is 8766 h (24 h × 365.25 d). *Q*_*in*_ is the indoor occupancy factor, *Q*_*out*_ is the outdoor occupancy factor, and they are 0.83 and 0.17, respectively, as reported by Ploykrathok et al. [30]. *R* is a reduction factor, the natural radionuclide is 1, and the artificial radionuclide is 0.43, as reported by Yoshida et al. [31].

## 3. Results and Discussion

### 3.1. Absorbed Dose Rate in Air and Dose Rate Map

**Figure 2:**
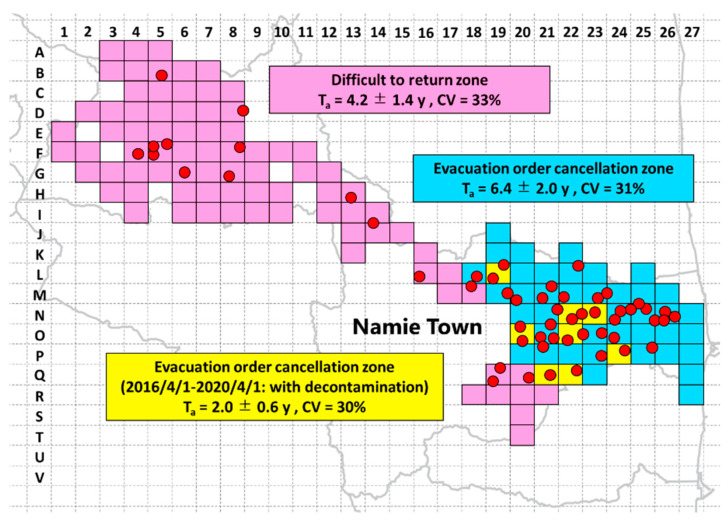
Area classification for which the apparent half-life was calculated, and the location of the monitoring posts. The red circles indicate the location of the monitoring posts used for the analysis, the blue mesh is the difficult-to-return zone, the pink mesh is the evacuation order cancellation zone where the radionuclides decontamination work was carried out before April 2016, and the green mesh is the evacuation order cancellation zone where the radionuclides decontamination work was carried out after April 2016. This map was drawn using a map created by Generic Mapping Tools [35].

The gamma-ray pulse-height distribution was measured over the entire accessible area of Namie Town and was developed using a response matrix to determine the absorbed dose rate in air. The absorbed dose rates in air of the natural radionuclides, artificial radionuclides, and their totals are 15–68, 14–11,861, and 47–11,900 nGy h^−1^, respectively. The total absorbed dose rate in air obtained in this study is almost in agreement with the 0.041–11 µGy h^−1^ measured by Shiroma et al. [21]. The absorbed dose rates in air of natural radionuclides, artificial radionuclides, and their totals in the evacuation order cancellation zone are 19–51, 14–2010, and 47–2040 nGy h^−1^, respectively. The natural, artificial, and their total absorbed dose rates in air in the difficult-to-return zone are 15–68, 140–11,861, and 186–11,900 nGy h^−1^, respectively. The radioactivity ratios of cesium (^134^Cs/^137^Cs) released from Units 1, 2, and 3 of the FDNPP were reported to be 0.941, 1.082, and 1.046, respectively [32]. This radioactivity ratio is evaluated as the value as of 11 March 2011. As a result of estimating ^134^Cs/^137^Cs as of March 2011 for the measured data, the median (range) was 1.07 (1.04–1.09), and it was confirmed that ^134^Cs and ^137^Cs were released from FDNPP. The apparent half-life was calculated by analyzing the datasets of 55 monitoring posts installed in Namie Townin order to time-correct the measured absorbed dose rate in air. A total of 32 of them were located in areas exceeding 1.0 µGy h^−1^ as of April 2016. 10 of them were located in areas exceeding 1.0 µGy h^−1^ as of April 2020. The mean ± standard deviation, coefficient of variation, and median (range) of apparent half-lives in the difficult-to-return zone are 4.2 ± 1.4 y, 33%, and 4.7 (4.0–4.8) y, respectively (Appendix A Table A1). Considering that the half-life of ^137^Cs is approximately 30 years, the reason why the apparent half-life is shortened is seemingly strongly influenced by diffusion due to environmental factors. The mean ± standard deviation, coefficient of variation, and median (range) of the apparent half-life in the evacuation order cancellation zone are 4.8 ± 2.7 y, 56%, and 4.7 (2.3–6.7) y, respectively. It was found that there are variations in the areas where residence is allowed. The apparent half-life was calculated using the data from 1 April 2016 to 1 April 2020. A detailed review of the data for each monitoring post revealed that some areas were decontaminated after April 2016, and some were decontaminated prior to that date [33]. The implementation of artificial decontamination contributes to rapid dose reduction and significantly shortens the apparent half-life. Therefore, the evacuation order cancellation zone was further divided into areas where decontamination was conducted before, and on and after, April 2016, and the apparent half-life was analyzed. Figure 2 indicates the difficult-to-return zone, evacuation order cancellation zone decontaminated before April 2016, and evacuation order cancellation zone decontaminated on, and after, April 2016 areas. The mean ± standard deviation, coefficient of variation, and median (range) of the apparent half-life in the evacuation order cancellation zone are 6.4 ± 2.0 y, 31%, and 6.1 (5.0–7.5) y, respectively (Appendix A Table A2). Conversely, the mean value ± standard deviation, coefficient of variation, and median (range) of the apparent half-life limited to the zones where decontamination was completed after 1 April 2016, are 2.0 ± 0.6 y, 30%, 1.8. (1.6–2.3) y, respectively (Appendix A Table A3). A significant difference test was performed using the Mann–Whitney *U* test for the apparent half-life of the evacuation order cancellation zone decontaminated before, and on and after, April 2016. It was confirmed there was a significant difference between the two groups (*p*-value < 3.8 × 10^–7^). This result demonstrates that the implementation of decontamination significantly contributes to the reduction of the ambient dose equivalent rates from artificial radionuclides. In addition, it was found that the evacuation order cancellation zone can be evaluated with a fluctuation of approximately 30%, by dividing it into two areas for the calculations. This coefficient of variation is significantly lower than when the evacuation order cancellation zone was not divided into two. In addition, a significant difference in apparent half-life was determined using the Mann–Whitney *U* test for the difficult-to-return zone and evacuation order cancellation zone decontaminated before April 2016, for the difficult-to-return zone and the evacuation order cancellation zone decontaminated on and after April 2016. The *p*-values are 6.9 × 10^−4^ and 9.5 × 10^−4^, respectively, confirming that there is a significant difference in distribution. Hayes et al. reported that the effective half-life of radiocesium in the environment was 7.8 years as a theoretical value and 3.2 years as a measured value [34]. Table 2 shows a comparison of the apparent half-life calculated in this study, the previously reported effective half-life, and the theoretical half-life.

**Figure 3:**
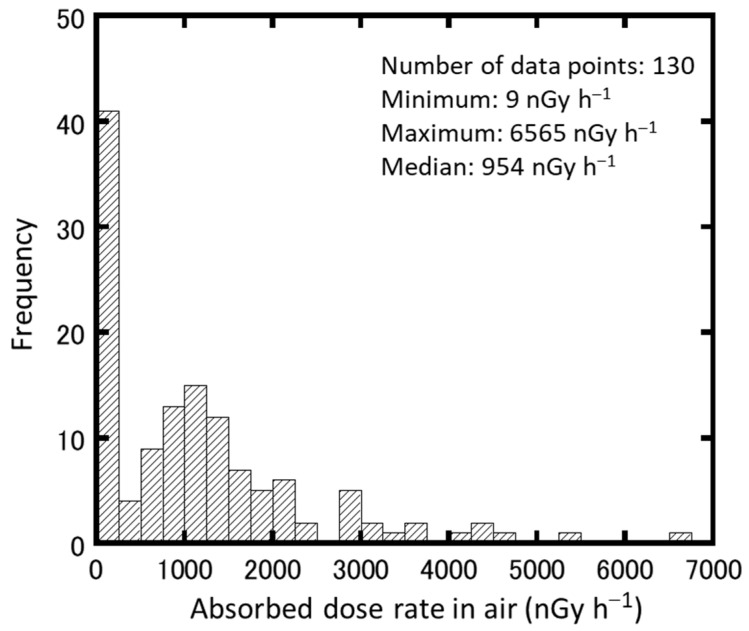
Histogram of absorbed dose rate in air of artificial radionuclides corrected as of 1 April 2020.

The measured data of absorbed dose rates in air from artificial radionuclides were corrected to the values as of 1 April 2020 using different apparent half-lives for each of the three areas (Appendix B). The median (range) is shown in Table 3, and the distribution of the absorbed dose rate in air of the artificial radionuclides collected as of 1 April 2020 is shown in Figure 3.

**Figure 4:**
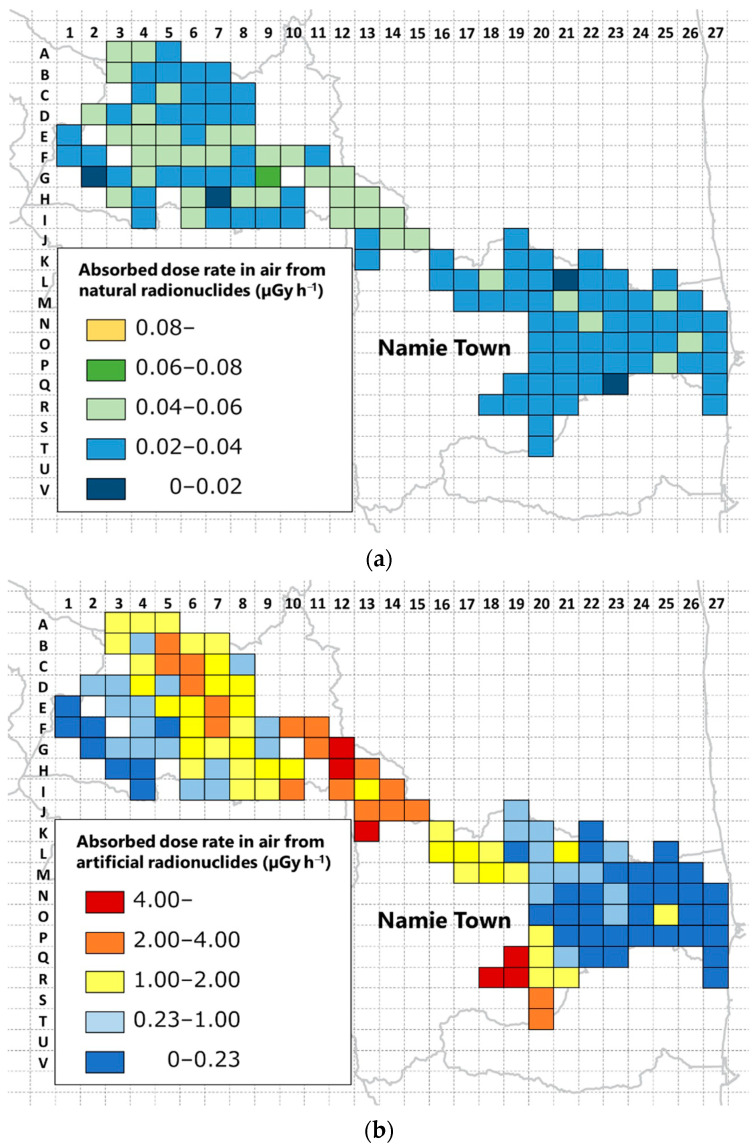
(**a**) Map of absorbed dose rate in air derived from natural radionuclides and (**b**) map of absorbed dose rate in air derived from artificial radionuclides. This map was drawn using a map created by Generic Mapping Tools [35].

A significant difference test was performed using the Mann–Whitney *U* test on the absorbed dose rates in the air from artificial radionuclides in the evacuation order cancellation zone and the difficult-to-return zone. It was confirmed that the two groups are significantly different (*p*-value = 6.0 × 10^−14^). The evacuation order cancellation zone is an area that the Japanese government has determined people can live in because it has been confirmed that the ambient dose equivalent rate has decreased [6]. In contrast, the difficult-to-return zone is an area where the annual cumulative dose exceeds 50 mSv as of April 2012, and the annual cumulative dose may not fall below 20 mSv after five years have elapsed [5]. It was found that the absorbed dose rate in air remained high in the difficult-to-return zone nine years after the FDNPP accident. The mean ± standard deviation and median (range) of absorbed dose rates in air by natural radionuclides throughout Namie Town are 35 ± 10 and 34 (28–42) nGy h^−1^, respectively. The national average in Japan is reported to be 50 nGy h^−1^ [36]. It was found that the average value of Namie Town was 70% of the national average value. These data can be used for radiation risk communication. The absorbed dose rate maps (Figure 4a,b) were developed so that the absorbed dose rate in air could be visually understood by dividing it into natural and artificial radionuclides.

The activity concentrations of ^40^K, ^232^Th, and ^238^U are shown in Appendix B. When examining the absorbed dose rate in air from natural radionuclides (Figure 4a), it can be seen that the eastern coastal area of Namie Town is less than 40 nGy h^−1^ in most areas. The range of activity concentrations of ^40^K, ^232^Th, and ^238^U in the evacuation order cancellation zone were 109–444, 9–32, and 9–34 Bq kg^−1^, respectively. Conversely, in the mountainous areas on the west side, there are many areas of 40 nGy h^−1^ or more. The range of activity concentrations of ^40^K, ^232^Th, and ^238^U in the difficult-to-return zone were 99–1830, 9–46, and 10–161 Bq kg^−1^, respectively. On the west side of Namie Town, where granite is widely distributed, the activity concentrations of ^40^K, ^232^Th, and ^238^U tended to be high [37]. When examining the absorbed dose rate in air from artificial radionuclides (Figure 4b), it can be seen that there is a clear difference between the coastal areas on the east side and the mountainous areas on the west side. This is a clear result of the evacuation order cancellation zone and the difficult-to-return zone. In the coastal area, decontamination was actively conducted in order to realize the return of evacuees, and the evacuation order was lifted in March 2017 [6]. In contrast, the mountainous area on the west side has many areas exceeding 1.0 µGy h^−1^, and is remains designated as a difficult-to-return zone. This result indicates that artificial decontamination activities contribute significantly to dose reduction. However, there were two meshes in the evacuation order cancellation area that exceeded 1.0 µGy h^−1^. Factors that increased the absorbed dose rate in air in this area include the presence of slopes composed of soil and the presence of localized forest areas in the city, such as bamboo groves. Slopes composed of soil have not been actively decontaminated because they may loosen the ground and cause sediment-related disasters. Local forest areas in the city, such as bamboo groves, are difficult to decontaminate by removing the upper part of the soil without cutting, which is a factor that increases the absorbed dose rate in air. However, local forests and slopes composed of soil do not always exist uniformly within a 1 km × 1 km mesh. In order to examine the variation of the measurement data in the mesh, the absorbed dose rate in air was additionally measured at 2–5 points in six out of the 130 meshes (Table 4). Although there are some fluctuations depending on the mesh, it was found that it is possible to evaluate with a volatility of approximately 50% or less. It was also determined that the volatility is not dose-dependent.

### 3.2. Estimating External Exposure Dose

Table 5 indicates the median (range) of the annual effective dose of external exposure calculated from the absorbed dose rate in the air. The annual effective doses of natural radionuclides in the evacuation order cancellation zone, difficult-to-return zone, and Namie Town as a whole are 0.12–0.33, 0.10–0.45, and 0.10–0.45 mSv, respectively, and their geometric mean (mean ± standard deviation) is 0.20 (0.20 ± 0.05), 0.24 (0.24 ± 0.06), and 0.22 (0.23 ± 0.06), respectively. The national average effective annual dose of ground gamma-rays in Japan is 0.33 mSv. It was found that the average value for the town of Namie is 70% of the national average [38,39]. The annual effective doses of external exposure to artificial radionuclides in the evacuation order cancellation zone, difficult-to-return zone, and entire Namie Town are 0.03–4.6, 0.23–19.6, and 0.03–19.6 mSv, respectively. The median annual external exposure effective dose from artificial radionuclides in the evacuation order cancellation zone (0.40 mSv) is 0.21 mSv, which differs from the median natural radionuclides (0.19 mSv). In contrast, the median annual external exposure effective dose from artificial radionuclides in the difficult-to-return zone (3.9 mSv) is 15.6 times higher than the median from natural radionuclides (0.25 mSv). A significant difference test was performed using the Mann–Whitney *U* test on the annual effective dose of external exposure from artificial radionuclides in the evacuation order cancellation zone and the difficult-to-return zone. The two groups have a statistically significant difference (*p*-value < 6.0 × 10^−^^14^). This difficult-to-return zone is an area where access to people is restricted. Cars are allowed on some sections, but the general public is still not allowed to stay for a long time [40]. Currently, in difficult-to-return zone, active decontamination is being carried out so that people can live. In the future, this artificial decontamination is expected to reduce the absorbed dose rate in air.

## 4. Conclusions

The absorbed dose rate in air was measured by discriminating between natural and artificial radionuclides in the entire area of Namie Town, an area affected by the FDNPP accident. The following results were obtained from this study:

1. From the measurements of ^134^Cs and ^137^Cs concentrations, it was confirmed that Namie Town was radioactively contaminated by artificial radionuclides from the FDNPP accident.
2. From the data of the monitoring posts installed in Namie Town, the median (range) of the apparent half-life of artificial radionuclides in the evacuation order cancellation zone decontaminated before April 2016, the evacuation order cancellation zone decontaminated after April 2016, and the difficult-to-return zone, is 6.4 ± 2.0, 2.0 ± 0.6, and 4.2 ± 1.4 y, respectively.
3. The median (range) of absorbed dose rates in the air from artificial radionuclides time-corrected as of 1 April 2020, using the apparent half-life are 133 (67–511) and 1306 (892–2081) nGy h^−1^ in the evacuation order cancellation zone and the difficult-to-return zone, respectively.
4. The median annual effective doses of external exposures from natural and artificial radionuclides are 0.19 and 0.40 mSv in the evacuation order cancellation zone and 0.25 and 3.9 mSv in the difficult-to-return zone.

Examination of the absorbed dose rate in the air from artificial radionuclides revealed a clear difference between the eastern coastal area and the western mountainous area. This result suggests that artificial decontamination activities contribute significantly to dose reduction. The distribution map of the absorbed dose rate in air measured in this study, and the information on the annual external exposure effective dose calculated by discriminating between natural and artificial radionuclides, are expected to be utilized for radiation risk communication.

## Figures and Tables

**Table 1:** The estimated value of the quantity of typical radionuclides released into the atmosphere by the Fukushima Daiichi Nuclear Power Plant (FDNPP) accident.

The Estimated Value of the Quantity of Radionuclides Released into the Atmosphere (Bq)							
^132^Te	^131^I	^132^I	^133^I	^133^Xe	^134^Cs	^136^Cs	^137^Cs
2.9 × 10^16^	1.2 × 10^17^	2.9 × 10^16^	9.6 × 10^15^	7.3 × 10^18^	9.0 × 10^15^	1.8 × 10^15^	8.8 × 10^15^

**Table 2:** Comparison of the half-life of radiocesium in the environment.

Apparent Half-Life of Radiocesium in the Environment (y)				
Evacuation Order Cancellation Zone		Difficult-to-Return Zone	Previously Reported Value [34]	Theoretical Value [34]
Decontaminated before April 2016	Decontaminated on and after April 2016			
6.4	2.0	4.2	3.2	7.8

**Table 3:** Median (range) estimated absorbed dose rate in air as of 1 April 2020.

	Absorbed Dose Rate in air as of 1 April 2020 (nGy h^−1^)	
	Evacuation Order Cancellation Zone	Difficult-to-Return Zone
Natural radionuclides	28 (25–35)	37 (30–45)
Artificial radionuclides	133 (67–511)	1306 (892–2081)
Total	161 (995–81)	1340 (921–2124)

**Table 4:** Evaluation of variation of measurements data in a 1 km × 1 km mesh.

Mesh Code	Number of Measurements	Absorbed Dose Rate in Air		
		Average ± Standard Deviation (nGy h^−1^)	Standard Error (nGy h^−1^)	Coefficient of Variation
F5	4	1118 ± 84	42	8%
L22	3	126 ± 33	19	26%
L23	6	312 ± 147	60	47%
M22	5	227 ± 83	37	37%
M24	4	156 ± 14	7	9%
N23	3	147 ± 44	25	30%

**Table 5:** Estimated annual external exposure effective dose.

	Median (Range) Annual External Exposure Effective Dose (mSv)	
	Evacuation Order Cancellation Zone	Difficult-to-Return Zone
Natural radionuclides	0.19 (0.16–0.23)	0.25 (0.20–0.29)
Artificial radionuclides	0.40 (0.20–1.5)	3.9 (2.7–6.2)
Total	0.55 (0.39–1.7)	4.1 (2.9–6.5)

## Appendix A

**Table A1:** Calculation table of apparent half-life in the difficult-to-return zone.

Mesh Code	Ambient Dose Equivalent Rate (µSv h^−1^)		Apparent Half-Life (y)
	As of 1 April 2016	As of 1 April 2020	
B5	4.2	2.4	4.7
D8	2.4	1.3	4.5
F4	1.2	0.64	4.7
F5	0.96	0.60	5.8
F5	4.9	0.70	1.4
F5	2.1	0.44	1.8
F8	3.6	2.0	4.6
G6	2.3	1.3	5.1
G8	1.7	0.88	4.2
H13	5.4	3.2	5.2
J14	6.4	3.2	4.0
L16	1.0	0.62	5.6
M18	3.6	2.0	4.8
Q19	2.2	1.2	4.7
Q19	11.8	5.7	3.9
Q20	4.7	0.69	1.4

**Table A2:** Calculation table of apparent half-life in the evacuation order cancellation zone where decontamination was conducted before 1 April 2016.

Mesh Code	Ambient Dose Equivalent Rate (µSv h^−1^)		Apparent Half-Life (y)
	As of 1 April 2016	As of 1 April 2020	
L18	1.5	0.76	3.9
L19	3.2	1.9	5.0
L22	0.40	0.28	8.2
M19	2.2	1.2	4.7
M20	0.59	0.41	7.5
M21	1.1	0.60	4.4
M21	0.38	0.24	6.1
M22	0.41	0.32	10.5
M23	0.25	0.18	8.8
M23	0.16	0.11	8.1
N22	0.88	0.38	3.3
N24	0.22	0.14	5.9
N24	0.19	0.14	10.1
N24	0.12	0.07	5.9
N25	0.25	0.15	5.5
N25	0.08	0.06	7.4
N25	0.10	0.07	6.2
N26	0.21	0.13	6.1
N26	0.13	0.09	6.4
N26	0.09	0.06	7.0
O23	0.46	0.24	4.2
O24	0.23	0.17	9.2
P21	0.64	0.30	3.6
P23	1.6	0.97	5.7
P25	0.16	0.11	7.1

**Table A3:** Calculation table of apparent half-life in the evacuation order cancellation zone where decontamination was conducted on, and after, 1 April 2016.

Mesh Code	Ambient Dose Equivalent Rate (µSv h^−1^)		Apparent Half-Life (y)
	As of 1 April 2016	As of 1 April 2020	
L19	1.2	0.36	2.3
N21	3.2	0.39	1.3
N22	2.5	0.26	1.2
N23	1.0	0.25	1.9
O20	2.1	0.47	1.8
O20	2.7	0.55	1.7
O21	1.2	0.26	1.8
O21	1.7	0.36	1.8
O21	1.2	0.34	2.3
O22	1.3	0.19	1.4
O22	0.58	0.27	3.6
P24	1.6	0.28	1.6
Q21	1.3	0.38	2.3

## Appendix B

**Table A4:** Measured absorbed dose rate in air from natural and artificial radionuclides, estimated absorbed dose rate in air from artificial radionuclide as of 1 April 2020, and activity concentrations of natural radionuclides.

Mesh Code	Measuring Date	Absorbed Dose Rate in Air (nGy h^−1^)			^40^K (Bq kg^−1^)	^232^Th (Bq kg^−1^)	^238^U (Bq kg^−1^)
		Artificial Radionuclides	Artificial Radionuclides as of 1 April 2020	Natural Radionuclides			
A3	2017/8/23	1620	1048	50	419	32	28
A4	2018/9/10	1320	1017	54	428	39	29
A5	2018/9/10	1600	1233	28	244	21	13
B3	2017/8/23	2230	1442	51	477	27	28
B4	2017/8/23	1370	886	26	248	15	14
B5	2017/11/1	3578	2390	22	273	18	11
B6	2017/8/23	2990	1934	33	262	22	19
B7	2017/11/1	1760	1175	22	178	12	14
C4	2017/8/23	1850	1196	23	139	16	15
C5	2017/8/23	3280	2121	47	431	22	28
C6	2017/11/1	3020	2017	37	354	22	19
C7	2017/11/3	1960	1310	38	286	22	25
C8	2017/11/1	1360	908	28	244	15	17
D2	2017/8/24	1230	796	49	400	28	30
D3	2017/8/24	1240	802	37	382	17	21
D4	2017/11/1	2840	1897	44	382	23	26
D5	2017/8/23	1520	983	39	363	18	23
D6	2017/11/1	3741	2498	29	317	22	15
D7	2017/11/1	2160	1442	32	311	17	17
D8	2017/11/1	1950	1302	30	288	13	18
E1	2017/8/24	239	155	36	314	18	22
E3	2017/8/24	811	525	41	351	25	23
E4	2017/8/24	954	617	41	367	22	24
E5	2017/8/23	2300	1487	45	391	22	28
E6	2017/11/1	2670	1783	35	300	16	23
E7	2017/11/1	3120	2083	43	407	25	22
E8	2017/11/1	2200	1469	50	502	27	25
F1	2017/8/24	305	197	30	302	16	15
F2	2017/8/25	246	159	29	252	17	17
F4	2017/8/24	1200	776	53	419	29	34
F5	2016/9/15	140	77	46	400	19	31
F6	2016/9/15	1980	1095	44	391	20	28
F7	2017/11/3	3010	2012	48	484	22	27
F8	2017/11/3	1770	1183	23	213	13	13
F9	2017/11/3	1060	709	48	428	22	30
F10	2017/11/3	5335	3566	45	545	42	25
F11	2017/11/3	5412	3618	38	530	37	21
G2	2017/8/24	255	165	17	143	10	10
G3	2017/8/24	376	243	31	216	19	20
G4	2017/8/24	891	576	45	407	22	27
G5	2017/8/25	1230	796	30	263	15	18
G6	2017/8/25	2380	1541	28	242	17	16
G7	2017/11/2	1930	1289	36	326	17	22
G8	2017/11/2	2080	1390	37	323	17	23
G9	2017/11/2	913	610	68	628	26	46
G11	2017/11/3	3100	2072	47	477	27	22
G12	2018/5/16	5890	4303	40	545	42	22
H3	2017/8/24	315	204	45	407	26	24
H4	2017/8/24	151	98	36	388	20	17
H6	2018/9/10	1310	1010	42	339	28	23
H7	2017/8/25	1430	926	15	99	11	9
H8	2017/11/2	1690	1129	45	437	16	30
H9	2017/11/2	2090	1396	43	348	22	28
H10	2017/11/2	2360	1577	32	304	18	16
H12	2018/5/16	6466	4723	44	659	45	25
H13	2016/9/16	5306	2935	54	678	45	31
I4	2017/8/24	207	134	28	251	15	16
I6	2018/9/10	1130	871	45	323	35	24
I7	2017/8/25	1220	790	37	330	18	23
I8	2017/11/2	1560	1042	38	348	15	24
I9	2017/11/2	1640	1096	37	333	15	24
I10	2017/11/2	3190	2131	34	407	15	15
I12	2018/5/16	4278	3125	52	582	36	30
I13	2018/9/11	2470	1904	41	354	31	19
I14	2018/5/16	3788	2767	52	447	35	30
J13	2018/5/16	3799	2775	31	336	24	16
J14	2018/5/16	4369	3192	41	459	28	23
J15	2018/5/16	3898	2847	42	394	22	24
J19	2017/12/22	925	724	23	208	15	12
K13	2018/5/16	5721	4179	39	502	39	22
K16	2018/9/10	1570	1210	40	308	33	19
K19	2017/12/22	595	466	21	199	13	10
K20	2017/12/22	1120	876	30	311	16	15
K22	2019/11/14	179	172	26	205	18	14
L16	2018/5/16	2490	1819	37	367	20	19
L17	2018/5/16	2090	1527	40	373	23	21
L18	2018/9/11	1510	1277	46	382	32	24
L19	2017/12/22	231	104	28	290	14	14
L20	2017/12/22	1100	861	24	189	13	15
L21	2017/12/22	1950	1526	19	109	13	13
L22	2017/12/22	147	115	28	258	16	15
L23	2018/12/26	285	249	30	265	20	15
L25	2018/12/26	89	78	22	169	14	13
M17	2018/5/16	1720	1256	24	220	16	11
M18	2018/5/16	2120	1549	33	281	21	18
M19	2016/9/16	1510	1031	26	181	16	16
M20	2017/12/22	1260	986	27	137	21	18
M21	2017/12/22	343	268	44	311	28	27
M22	2016/9/16	423	289	23	222	12	13
M23	2017/12/23	242	189	35	360	17	18
M24	2018/12/26	105	92	32	258	18	20
M25	2018/12/26	35	31	51	339	34	32
M26	2017/12/23	33	26	39	388	17	23
N20	2017/12/22	854	668	24	197	14	14
N21	2017/12/22	174	136	32	305	19	15
N22	2018/5/16	141	73	47	444	20	29
N23	2017/12/22	1460	654	38	413	15	21
N24	2016/9/16	14	9	39	336	20	24
N25	2016/9/15	67	46	28	298	12	16
N26	2018/12/26	76	67	31	244	16	20
N27	2017/12/23	27	21	39	379	18	22
O20	2017/12/22	135	60	26	260	15	12
O21	2017/12/22	120	94	27	272	15	14
O22	2017/12/22	306	137	23	230	12	13
O23	2017/12/23	398	312	34	298	19	19
O24	2017/12/23	231	181	36	388	18	18
O25	2017/12/23	1690	1323	34	257	20	22
O26	2018/12/26	182	159	44	416	22	25
O27	2018/12/26	19	17	32	285	20	17
P20	2017/8/26	2010	1519	25	184	15	16
P21	2017/12/22	100	78	24	242	14	11
P22	2017/12/22	53	41	34	357	19	16
P23	2018/12/26	149	130	21	224	10	11
P24	2016/9/17	563	162	28	236	20	13
P25	2017/12/23	39	30	46	407	31	23
P26	2018/12/26	76	66	40	360	22	22
P27	2018/12/26	20	18	27	254	14	15
Q19	2016/9/17	9604	5316	26	1260	91	13
Q20	2018/9/10	1480	1141	30	257	18	16
Q21	2017/8/26	1620	648	27	260	15	15
Q22	2018/9/11	206	119	28	257	15	15
Q23	2018/9/11	98	83	20	181	14	9
Q27	2018/12/26	61	53	22	225	9	13
R18	2018/9/10	5560	4285	30	416	44	16
R19	2016/9/17	11861	6565	39	1830	161	22
R20	2017/8/26	1650	1069	30	280	18	15
R21	2017/8/26	2390	1548	21	202	15	9
R27	2018/12/26	101	88	30	266	16	18
S20	2017/8/26	5342	3460	28	360	30	15
T20	2017/8/26	4269	2764	21	342	24	11

## References

[1] Yaneza J., Kuznetsova M., Souto-Iglesiasb A. (2015). An analysis of the hydrogen explosion in the Fukushima-Daiichi accident. Int. J. Hydrogen Energy.

[2] Tominaga T., Hachiya M., Tatsuzaki H., Akashi M. (2014). The Accident at the Fukushima Daiichi Nuclear Power Plant in 2011. Health Phys..

[3] United Nations Scientific Committee on the Effects of Atomic Radiation (2014). UNSCEAR 2013 Report Annex A: Levels and Effects of Radiation Exposure Due to the Nuclear Accident after 2011 Great East-Japan Earthquake and Tsunami.

[4] Prime Minister’s Office of Japan Evacuation Order. (Tentative Translation). http://www.kantei.go.jp/jp/kikikanri/jisin/20110311miyagi/20110312siji11.pdf.

[5] Fukushima Revitalization Station Transition of Evacuation Designated Zones. https://www.pref.fukushima.lg.jp/site/portal-english/en03-08.html.

[6] Namie Town About Area Reorganization and Evacuation Order Cancellation. (Tentative Translation). https://www.town.namie.fukushima.jp/soshiki/2/13457.html.

[7] Namie Town Now in Namie Town. (Tentative Translation). https://www.town.namie.fukushima.jp/.

[8] Fukushima Revitalization Station The Situation in Namie Town. (Tentative Translation). https://www.pref.fukushima.lg.jp/site/portal/26-11.html.

[9] Takebayashi Y., Lyamzina Y., Suzuki Y., Murakami M. (2017). Risk Perception and Anxiety Regarding Radiation after the 2011 Fukushima Nuclear Power Plant Accident: A Systematic Qualitative Review. Int. J. Environ. Res. Public Health.

[10] Kudo H., Tokonami S., Hosoda M., Iwaoka K., Kasai Y. (2016). Understanding of Basic Knowledge on Radiation among General Public –Comparison of Residents Participated in the Same Seminar in between Namie Town and Three Cities in Aomori Prefecture–. Jpn. J. Health Phys..

[11] Saito K., Tanihara I., Fujiwara M., Saito T., Simoura S., Otsuka T., Onda Y., Hoshi M., Ikeuchi Y., Takahashi F. (2015). Detailed deposition density maps constructed by large-scale soil sampling for gamma-ray emitting radioactive nuclides from the Fukushima Dai-ichi Nuclear Power Plant accident. J. Environ. Radioact..

[12] Hosoda M., Tokonami S., Sorimachi A., Monzen S., Osanai M., Yamada M., Kashiwakura I., Akiba S. (2011). The time variation of dose rate artificially increased by the Fukushima nuclear crisis. Sci. Rep..

[13] Andoh M., Nakahara Y., Tsuda S., Yoshida T., Takahashi F., Mikami S., Kinouchi N., Sato T., Tanigaki M., Takemiya K. (2015). Measurement of air dose rates over a wide area around the Fukushima Dai-ichi Nuclear Power Plant through a series of car-borne surveys. J. Environ. Radioact..

[14] Akahane K., Yonai S., Fukuda S., Miyahara N., Yasuda H., Iwaoka K., Matsumoto M., Fukumura A., Akashi M. (2013). NIRS external dose estimation system for Fukushima residents after the Fukushima Dai-ichi NPP accident. Sci. Rep..

[15] Ishikawa T., Yasumura S., Ozasa K., Kobashi G., Yasuda H., Miyazaki M., Akahane K., Yonai S., Ohtsuru A., Sakai A. (2015). The Fukushima Health Management Survey: Estimation of external doses to residents in Fukushima Prefecture. Sci. Rep..

[16] Tokonami S., Hosoda M., Akiba S., Sorimachi A., Kashiwakura I., Balonov M. (2012). Thyroid doses for evacuees from the Fukushima nuclear accident. Sci. Rep..

[17] Hayano R.S., Tsubokura M., Miyazaki M., Satou H., Sato K., Masaki S., Sakuma Y. (2013). Internal radiocesium contamination of adults and children in Fukushima 7 to 20 months after the Fukushima NPP accident as measured by extensive whole-body-counter surveys. Proc. Jpn. Acad. Ser. B.

[18] Hosoda M., Tokonami S., Akiba S., Kurihara O., Sorimachi A., Ishikawa T., Momose T., Nakano T., Mariya Y., Kashiwakura I. (2013). Estimation of internal exposure of the thyroid to ^131^I on the basis of ^134^Cs accumulated in the body among evacuees of the Fukushima Daiichi Nuclear Power Station accident. Environ. Int..

[19] Kim E., Kurihara O., Tani K., Ohmachi Y., Fukutsu K., Sakai K., Akashi M. (2016). Intake ratio of ^131^I to ^137^Cs derived from thyroid and whole-body doses to Fukushima residents. Radiet. Prot. Dosim..

[20] Nuclear Regulation Authority Japan Monitoring Information of Environmental Radioactivity Level. (Tentative Translation). http://radioactivity.nsr.go.jp/map/ja/.

[21] Shiroma Y., Hosoda M., Iwaoka K., Hegedűs M., Kudo H., Tsujiguchi T., Yamaguchi M., Akata N., Kashiwakura I., Tokonami S. (2019). Changes of Absorbed Dose Rate in Air by CAR-BORNE Survey in Namie Town, Fukushima Prefecture After the Fukushima Daiichi Nuclear Power Plant Accident. Radiet. Prot. Dosim..

[22] EMF Japan Co Product Overview of EMF211 Type Gamma Ray Spectrometer. (Tentative Translation). https://www.emf-japan.com/emf/img/PDF/emf211-survey.pdf.

[23] Radiation Earth Science Laboratory Introduction of 49x49 Response Matrix for Environment Gamma Ray Analysis. (Tentative Translation). http://www1.s3.starcat.ne.jp/reslnote/YONQ.pdf.

[24] Minato S. (2001). Diagonal elements fitting technique to improve response matrixes for environmental gamma ray spectrum unfolding. Radioisotopes.

[25] International Commission on Radiation Unites and Measurements (1994). ICRU Report 53: Gamma-Ray Spectrometry in the Environment.

[26] Sanada Y., Urabe Y., Sasaki M., Ochi K., Torii T. (2018). Evaluation of ecological half-life of dose rate based on airborne radiation monitoring following the Fukushima Dai-ichi nuclear power plant accident. J. Environ. Radioact..

[27] Kinase S., Takahashi T., Saito K. (2017). Long-term predictions of ambient dose equivalent rates after the Fukushima Daiichi nuclear power plant accident. J. Nucl. Sci. Technol..

[28] Moriuchi S., Tsutsumi M., Saito K. (1990). Examination on Conversion Factors to Estimate Effective Dose Equivalent from Absorbed Dose in Air for Natural Gamma Radiations. Jpn. J. Health Phys..

[29] Omori Y., Wakamatsu H., Sorimachi A., Ishikawa T. (2016). Radiation survey on Fukushima Medical University premises about four years after the Fukushima nuclear disaster. Fukushima J. Med. Sci..

[30] Ploykrathok T., Ogura K., Shimizu M., Hosoda M., Shiroma Y., Kudo H., Tamakuma Y., Tokonami S. (2020). Estimation of annual effective dose in Namie Town, Fukushima Prefecture due to inhalation of radon and thoron progeny. Radiat. Emer. Med..

[31] Yoshida H., Hosoda M., Kanagami T., Uegaki M., Tashima H. (2014). Reduction factors for wooden houses due to external γ-radiation based on in situ measurements after the Fukushima nuclear accident. Sci. Rep..

[32] Nishizawa Y., Yoshida M., Sanada Y., Torii T. (2016). Distribution of the ^134^Cs/^137^Cs ratio around the Fukushima Daiichi nuclear power plant using an unmanned helicopter radiation monitoring system. J. Nucl. Sci. Technol..

[33] Ministry of the Environment Decontamination Information Site—Specified Reconstruction and Regeneration Base [Namie Town]- (Tentative Translation). http://josen.env.go.jp/kyoten/namie/.

[34] Hayes J.M., Johnson T.E., Anderson D., Nanba K. (2020). Effective Half-life of ^134^Cs and ^137^Cs in Fukushima Prefecture When Compared to Theoretical Decay Models. Health Phys..

[35] Wessel P., Smith W.H.F. (1991). Free software helps map and display data. Trans. Am. Geophys. Union.

[36] Furukawa M., Shingaki R. (2012). Terrestrial gamma radiation dose rate in Japan estimated before the 2011 Great East Japan Earthquake. Radiat. Emerg. Med..

[37] Geological Survey of Japan Geology of Namie, Iwaki and Tomioka Areas. (Tentative Translation). https://www.gsj.jp/data/50KGM/PDF/GSJ_MAP_G050_07046_1994_D.pdf.

[38] Omori Y., Hosoda M., Takahashi F., Sanada T., Hirao S., Ono K., Furukawa M. (2020). Japanese population dose from natural radiation. J. Radiol. Prot..

[39] Hosoda M., Tokonami S., Furukawa M. (2012). Dose assessment on natural radiation, natural radionuclide, and artificial radionuclide released by the Fukushima nuclear accident. Radiat. Biol. Res. Commun..

[40] Ministry of Economy, Trade and Industry Standards for Temporary Entry into Difficult-to-Return Zone. (Tentative Translation). https://www.meti.go.jp/earthquake/nuclear/kinkyu/hinanshiji/pdf/190905_zissikizyun2.pdf.

